# Mucinous Tubular and Spindle Cell Renal Cell Carcinoma (MTSC-RCC) with an Unusual Presentation in a Child

**DOI:** 10.15586/jkcvhl.v9i4.223

**Published:** 2022-10-29

**Authors:** Kanika Sharma, Anjan Dhua, Sandeep Agarwala, Seema Kaushal

**Affiliations:** 1 Department of Pediatric Surgery, All India Institute of Medical Sciences, New Delhi, India;; 2 Department of Pathology, All India Institute of Medical Sciences, New Delhi, India

**Keywords:** children, mucinous tubular and spindle cell carcinoma, renal cell carcinoma

## Abstract

Mucinous tubular and spindle cell renal cell carcinoma (MTSC-RCC) is a rare but favorable variant of renal cell carcinoma, predominantly found in adults. Complete surgical excision is the treatment of choice. We are reporting an intriguing case of bilateral MTSC-RCC in a 13-year-old-boy with rapid disease progression, leading to metastatic disease and subsequent demise of the child.

## Introduction

The incidence of childhood renal tumors is around 7%, with 90% of them being Wilms tumor. Amongst the non-Wilms renal tumors, clear-cell sarcoma and malignant rhabdoid tumor of the kidney are the commonest. Renal cell carcinoma (RCC) comprises 1% of all pediatric renal tumors and is generally seen in the adolescent age group (1). Mucinous tubular and spindle cell renal cell carcinoma (MTSC-RCC), a rare variant of RCC, found predominantly amongst adults, has been described with an indolent course and favorable outcome after resection. We report a case of MTSC-RCC in a 13-year-old boy with an unusual clinical course with progressive disease. Bilateral involvement with MTSC-RCC has not been reported previously.

## Case Report

**Figure 1:**
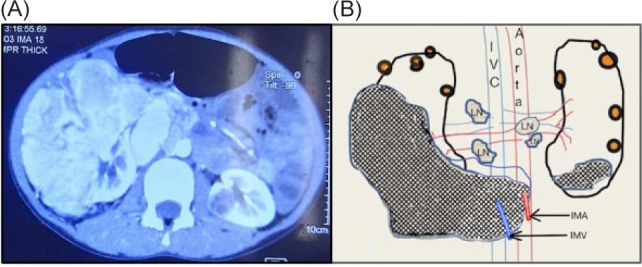
(A) CECT (axial section) demonstrating a heterogeneously enhancing in right kidney with foci of calcification. A small hypodense lesion is noted on lower pole of left kidney. (B) Diagrammatic representation of the overall CECT findings with the masses (cross-hatch), the involvement of the lymph nodes, and bilateral cortical cysts. IVC, inferior vena cava; IMA, inferior mesenteric artery; IMV, inferior mesenteric vein, and LN, lymph node.

**Figure 2:**
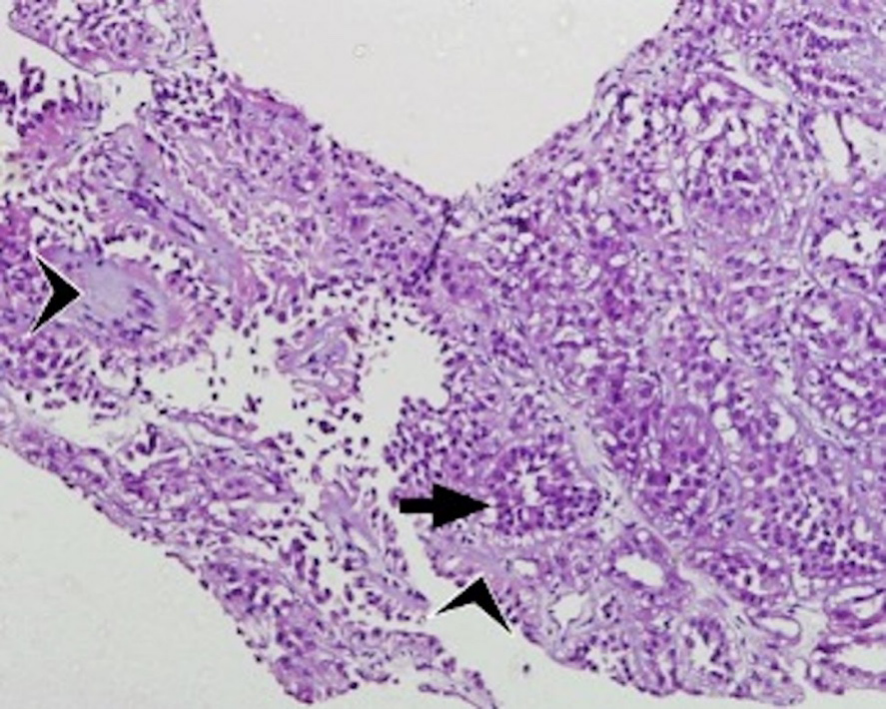
Tumor composed of tubules (arrow) with intervening mucoid matrix (arrowheads). H & E,10×.

**Figure 3:**
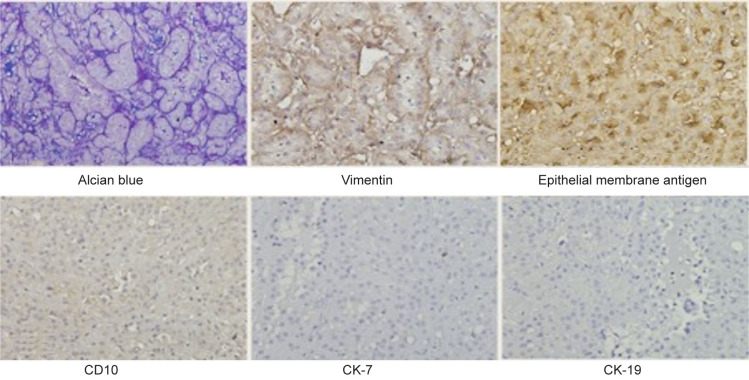
On immnunohistochemistry, the tumor characteristically shows the mucoid material is highlighted in blue on Alcian blue, and the tumor cells are immunopositive for vimentin, epithelial membrane antigen, and are negative for CD10, CK-7, and CK-19.

A 13-year-old boy presented with complaints of right flank mass with occasional dull, localized pain noted for 3 months. There was associated history of intermittent episodes of painless hematuria. There was no history of fever, urinary tract infection, anorexia, or weight loss. On physical examination, an 8 × 6 cm, firm, and mobile renal mass was found occupying the right lumbar, the right hypochondrium, the umbilical regions, and crossing the midline. The laboratory investigations were within normal limits. The ultrasonography was suggestive of a large (10.2 × 9.4 × 10.3 cm) nodular heteroechoic mass in the right kidney with calcifications and was invading the renal vessels. The abdomen’s contrast-enhanced computed tomography (CECT) revealed a 9.6 × 7.8 cm heterogeneously enhancing soft tissue density in the mid and lower body of the right kidney with foci of patchy calcifications. The mass was encasing the inferior mesenteric artery and vein. The left kidney had multiple hypodense lesions on the lower pole. There was a large left para-aortic lymph node. There were multiple cortical cysts bilaterally (Figure 1). There was no thrombus in the inferior vena cava. CECT chest was normal with no metastasis. An ultrasound-guided core needle biopsy was performed that demonstrated tumor cells composed of tubules with a mucoid matrix (Figure 2), which on immunohistochemistry was positive for vimentin and epithelial membrane antigen (Figure 3), suggestive of a mucinous tubular and spindle cell tumor variant of renal cell carcinoma. However, no high-grade mitotic activity, malignant features, or sarcomatoid histology could be appreciated in the sections studied.

The child underwent an exploratory laparotomy, which revealed an unresectable tumor mass because of encasement of the inferior vena cava (IVC) and aorta. Bilateral wedge biopsies were taken. The child had an uneventful postoperative hospital stay and was discharged. The histopathology report of the bilateral renal biopsies corroborated with the needle biopsy. As the parents were not keen on chemotherapy, the child was advised for metronomic therapy. After 3 months, a repeat radiological evaluation pointed out that the disease had progressed locally and systematically, with the involvement of the bilateral lungs and the liver. The child succumbed to progressive disease within 10 months of the initial presentation.

## Discussion

The MTSC-RCC is a rare variant of renal cell carcinoma. This entity has been recently identified as a separate variant of RCC and had been classified in the 2004 World Health Organization (WHO) tumor classification (2). MTSC-RCC is predominately seen in adult patients. The mean age of presentation is 53 years, with a female predominance of 2–4:1 (3). On careful review of the literature, the youngest patient of MTSC-RCC was found to be a 17-year-old adolescent (4). Thus, the present case should be considered the youngest. Most of these tumors are incidentally detected during imaging for unrelated causes; however, a small group of these patients may present with flank masses, hematuria, and abdominal pain, as seen in our case, where the predominant features were flank mass, pain, and hematuria. MTSC-RCC has been associated with nephrolithiasis and end-stage renal disease in some cases (4).

Radiologically, MTSC-RCC is usually an exophytic renal mass with an expansile growth pattern and heterogenous enhancement pattern on the CECT scan. It resembles the papillary RCC more than a clear-cell RCC. There can be features of calcification and intratumor bleed. The CECT, in our case, showed that the mass had most of these features with a heterogenous enhancement and foci of calcification as well. Few studies have shown that T2-weighted magnetic resonance imaging demonstrates high signal intensity with a low apparent diffusion coefficient of the tumor and helps differentiate cases from papillary renal cell carcinoma (5).

Grossly, the tumor is well-circumscribed, partially encapsulated, arising from the renal cortex. The cut surface is uniformly solid gray or pale yellow in color with a shiny mucoid surface (6). Microscopically, the tumor demonstrates an admixture of tubular and spindle cells in a mucinous stroma (4). Tumor cells have round, uniform, low-grade nuclei and scantly mildly eosinophilic cytoplasm. Extracellular blue-gray mucinous matrix is abundant (classical variant) or scanty (mucin-poor variant). However, few reports of high-grade cellular proliferation with cellular atypia, tumor necrosis, and high mitotic activity have been documented (7). The origin of MTSC-RCC is unclear. However, its positivity for epithelial markers such as epithelial membrane antigen, E-cadherin, CK8/18, CK19, and CK7 on immunohistochemistry indicates its epithelial origin. MTSC-RCC shows variable staining seen with vimentin, RCC markers, chromogranin A, synaptophysin, CD10, and CD15; however, it is negative for smooth muscle actin, WT1, CD117, P63, and CK20. The histopathology findings in our case corroborated with the classical variant of MTSC-RCC with the tumor demonstrating an admixture of tubular cells and spindle cells with an extracellular mucinous matrix. Immunohistochemistry revealed features that suggested an epithelial origin of the tumor (positive for Epithelial membrane antigen); in addition, the tissue showed positive staining for vimentin but was negative for CD 10. MTSC-RCC is a histopathologically heterogenous tumor with morphology, radiology, and immunohistology similar to papillary RCC, while it is a distinct entity genetically (8). The molecular and chromosomal abnormalities are characteristic of this tumor, irrespective of histological variations (3, 9). MTSC-RCC lack typical gain in chromosome 7 and 17 which differentiates it from other differential diagnosis of papillary RCC, clear-cell RCC, sarcomatoid RCC, smooth muscle tumors, metanephric adenoma, and inflammatory myofibroblastic tumor.

Like other renal cell carcinoma, surgical excision of MTSC-RCC remains the cornerstone of management without the need for adjuvant local therapy or chemotherapy. Although no established treatment protocols or trials have been drafted for progressive or metastatic disease, owing to its rarity, there are few reports of response to Sunitinib, Gemitcitabine, or immunotherapy (ipilimumab and nivolumab) in a case of metastatic MTSC-RCC (10–12). In our case, the intraoperative findings precluded a safe surgical excision, and hence we had to resort to alternate strategies. In the absence of any guidelines, we offered metronomic therapy (with Sunitinib or Nivolumab in adjusted lower dose than the maximum tolerated dose repetitively over a long period to treat cancers with fewer side effects as a palliative measure only) as the parents were reluctant to go for conventional chemotherapy of any kind. Due to the scarcity of literature on pediatric MTSC-RCC, the management and prognostication of this child were challenging.

The MTSC-RCC is a distinct variety of renal cell carcinoma. In its classical form, it’s a low-grade tumor with an indolent course. However, reports on associated sarcomatoid components have emerged, which are responsible for high-grade tumor with aggressive behavior with coexisting low-grade tumor components (9). In addition, there are reports of aggressive behavior of MTSC-RCC in cases of a mucin-poor variant. Few reports of tumor recurrences, metastasis (lymph nodes and distant), and mortality have been documented (4). Therefore, a long-term close follow-up is warranted.

The present report was unique in many ways. There have been no prior reports on bilateral renal involvement with MTSC-RCC, especially in a child. The unfavorable clinical course in the absence of aggressive or malignant pathological features was also intriguing. The unexpected aggressive clinical course could be attributed to a missed sarcomatoid foci or sarcomatoid degeneration developing later in the course of the disease.

## Conclusion

Although a rare variant of adult RCC, MTSC-RCC tumors can also be encountered in the pediatric age group, albeit rarely. Such tumors may affect the kidneys bilaterally and can have progressive disease with metastasis, despite the absence of suggestive aggressive pathological features, leading to a poor outcome.
